# Emotional expressions beyond facial muscle actions. A call for studying autonomic signals and their impact on social perception

**DOI:** 10.3389/fpsyg.2015.00711

**Published:** 2015-05-27

**Authors:** Mariska E. Kret

**Affiliations:** ^1^Brain and Cognition, Department of Psychology, University of AmsterdamAmsterdam, Netherlands; ^2^Amsterdam Brain and Cognition CenterAmsterdam, Netherlands

**Keywords:** affect, pupil size, facial reddening, tears, contagion, synchronization, impression formation

## Abstract

Humans are well adapted to quickly recognize and adequately respond to another’s emotions. Different theories propose that mimicry of emotional expressions (facial or otherwise) mechanistically underlies, or at least facilitates, these swift adaptive reactions. When people unconsciously mimic their interaction partner’s expressions of emotion, they come to feel reflections of those companions’ emotions, which in turn influence the observer’s own emotional and empathic behavior. The majority of research has focused on facial actions as expressions of emotion. However, the fact that emotions are not just expressed by facial muscles alone is often still ignored in emotion perception research. In this article, I therefore argue for a broader exploration of emotion signals from sources beyond the face muscles that are more automatic and difficult to control. Specifically, I will focus on the perception of implicit sources such as gaze and tears and autonomic responses such as pupil-dilation, eyeblinks and blushing that are subtle yet visible to observers and because they can hardly be controlled or regulated by the sender, provide important “veridical” information. Recently, more research is emerging about the mimicry of these subtle affective signals including pupil-mimicry. I will here review this literature and suggest avenues for future research that will eventually lead to a better comprehension of how these signals help in making social judgments and understand each other’s emotions.

## Introduction

Imagine how different life would be if we were unable to recognize another’s expressions and from that, infer or even *feel* how the other must be feeling? During interactions with others, we automatically make use of another’s facial expressions and bodily signals and use that information to contextualize what is being said. Often, we are not aware of being influenced by these signals, except when they are absent from a “conversation,” for example during email or phone (mis)communication. Modern communication media aim for making conversations as natural as possible. However, even a Skype-conversation which from all communication media best simulates a natural interaction, does not even approach the richness and quality of real face-to-face interactions. One key limitation of Skype is that it is impossible to make eye-contact. Making eye-contact over Skype would require both interaction partners to look into the camera (and miss out upon each other’s eyes). **Table 1** gives an overview of the various cues available in different communication mediums. In this review I will focus on the visual domain and on the face alone (columns E and F in **Table 1**) and explain how humans use a variety of sources from others’ facial signals during natural interactions.

**Table 1:** Cues available in different communication mediums.

Medium	(A) Literal Message	(B) Shared environment	(C) Auditory	(D) Other sensory cues	(E) Facial expression	(F) Eye contact	(G) Body language
Real life	✓	✓	✓	✓	✓	✓	✓
Skype	✓	–	✓	✓	✓	–	✓/–
Phone	✓	–	✓	–	–	–	–
Email	✓	–	–	–	–	–	–

*Depending on the type of communication medium, more is transmitted than just the literal, “cold” message (A). Apart from interfering visual or auditory delays, one large difference between real life interactions and interactions via other mediums is the being versus not being in the same environment, both in terms of time and place (B). In addition, during email contact, one misses out upon important auditory cues such as the tone of voice of the interaction partner, and his intonation or use of sarcasm which might change the meaning of the literal message (C). Apart from auditory cues, other sensory information such as smell and touch might impact on how the literal message is transferred and interpreted (D). The face, its expression and autonomic signals that are visible in it provide important cues about the emotional state of the interaction partner such as intensity or genuineness (E). Eye-contact is very important during interactions and is impossible via modern communication mediums (F). Body language and autonomic signals visible from the body reveal important emotional information (G)*.

Emotion processing is a broad and general term that refers to a complex of affective, behavioral and cognitive mechanisms that underlie our emotions. Given the impact of our emotions on a wide range of mental processes (e.g., perception, impression formation, decision making, memory) and manifest behaviors (e.g., helping or aggressive and abusive behavior), being able to recognize and regulate our emotions is of crucial importance and an essential feature of mental health (Kret and Ploeger, 2015).

During social interaction, interaction partners continuously express and regulate emotional states and simultaneously process affective cues expressed by the other. They orient to their partner’s tractable characteristics, such as facial or bodily features and emotion expressions. By attending to the stream of subtle dynamic facial reactions during an interaction, they “feel themselves into” the emotional landscapes inhabited by their partners; they rely on, and are influenced by implicit signals from their partner’s face that are autonomic and not under someone’s control, yet reflective of his or her emotions and intentions (Hatfield et al., 1994). This interchange of emotion processing influences impressions that are formed during a social interaction.

**FIGURE 1:**
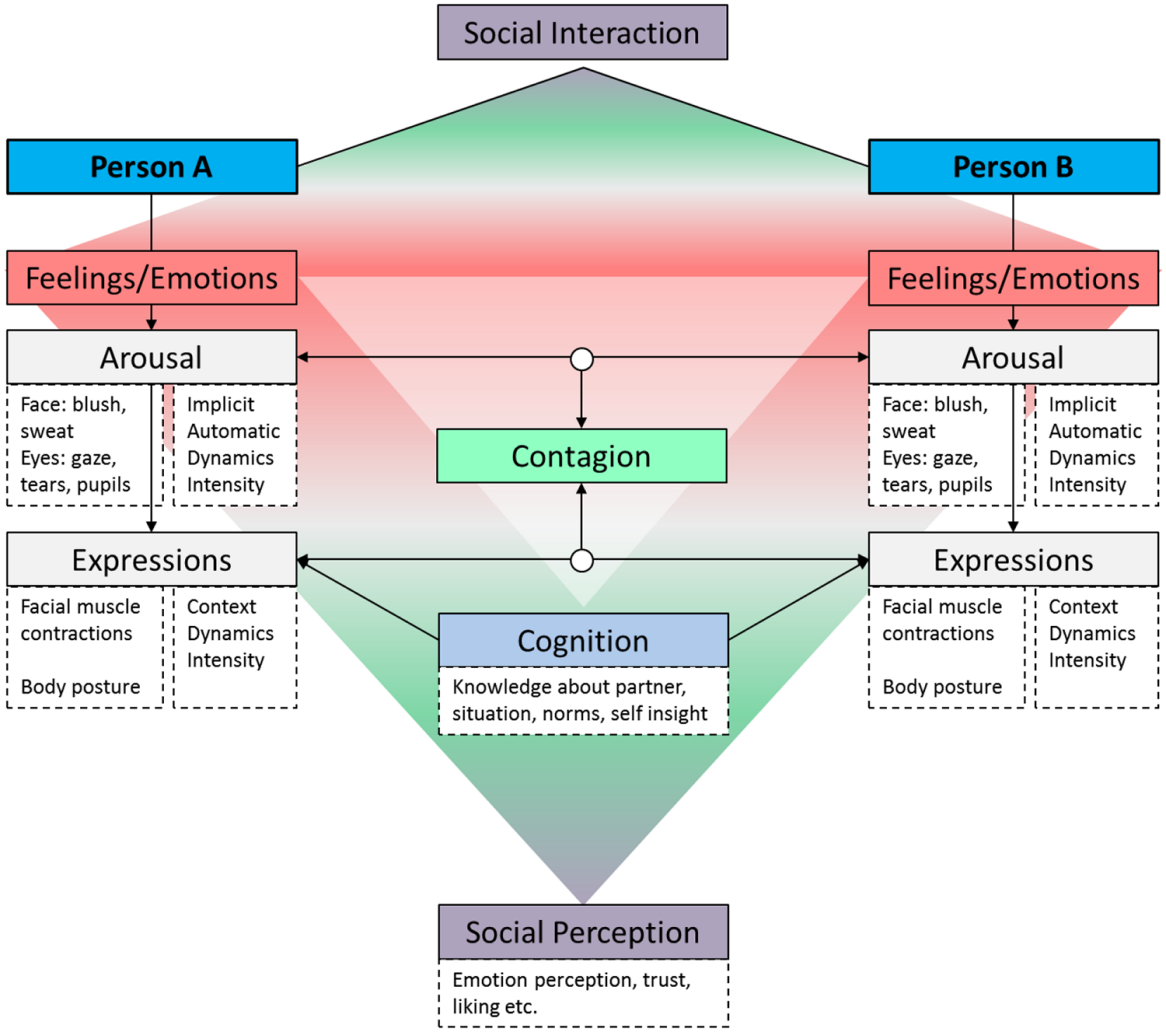
**Schematic representation of emotion processing during a social interaction**.

**Figure 1** shows how *emotions* that are expressed during a social interaction by Person A, through emotional *contagion*, influence the emotions and expressions of Person B. Person A and B not only mimic each other’s facial expression, they also link on the physiological level and without being aware of it, synchronize on the level of arousal. Whereas they from time to time may “force” social smiles when considered appropriate (this is where ‘*cognition*’ comes into play), they have no or very little control over their autonomic responses such as blushing, sweating and pupil dilation which may nonetheless spread to the other person. Emotions and feelings, the extent to which they are expressed and converged with, together with cognitive processes influence how the other person is perceived. I call this ‘*social perception*,’ which includes impressions, beliefs about how the other person is feeling, trust, liking etc.

The fact that emotions are not just expressed by the face and its muscle actions alone is often ignored in emotion perception research. In this article, I therefore argue for a broader exploration of emotion signals from sources beyond the face muscles that are more difficult to control. More specifically, I will focus on the perception of implicit sources that may be equally salient and familiar in daily life such as eye-gaze, pupil-dilation, eyeblinks, blushing and sweating. Although these cues may be subtle, they are visible to observers and may add to the perceived intensity of an emotional expression, or to its perceived genuineness. But prior to discussing these autonomic responses, I will review the literature on facial emotion perception.

### Emotions Shown by the Whole Face

Expressions of emotion have evolved not only to move blood and tears in the service of emotions, but also to provide us with a rich set of tools that help us communicate and signal the nature of our internal emotional experiences so that we can call on others empathy and receive help.

#### Emotions Expressed by Face Muscles

Humans are experts at processing faces. They can recognize the identity of 1000s of individual faces and in addition quickly decode a variety of emotional expressions. Like the face itself, facial expressions of emotion may be processed configurally, a processing style that presumably enables speed and efficiency (Tanaka and Farah, 1993). The hallmark of this processing routine is the inversion effect, i.e., the impaired recognition performance when a face is shown upside down (Yin, 1969; for a review, see Maurer et al., 2002). Inversion appears to weaken or remove emotion category boundaries along continua of morphed stimuli and it has therefore been argued that the categorization of facial expressions draws upon configural information (de Gelder et al., 1997). It has also been proposed that categorical processing can be based on features, for example for happy expressions with the salient mouth curvature feature or for fearful faces with the display of eye-white, and, thus, precede affective attribution at the stage of configural processing (Calvo et al., 2012). This might explain why inversion has no effect when facial expressions are employed in a visual search task (Lipp et al., 2009; Savage and Lipp, 2014).

The many distinct facial expressions of emotions can be accurately encoded with aid of the facial action coding system (FACS; Ekman and Friesen, 1978). This anatomically based system segments the visible effects of facial muscle activation into “action units” (AUs). Each AU relates to one or more facial muscles. FACS describes facial activity on the basis of 33 unique AUs. This system not only helped in developing standardized stimuli sets of facial expressions of emotion [for example JACFEE (Biehl et al., 1997) and Ekmans pictures (Ekman and Friesen, 1978)], but also resulted in the development of automated facial expression recognition software.

Emotional expressions, facial expressions included, are highly contagious. It has been suggested that facial mimicry, i.e., the imitation of others’ facial displays by an observer, plays an important role in the communication of affective states. Evidence exists that mimicry accompanies the perception of a facial expression (Dimberg, 1982; Bush et al., 1989). The mere observation of a facial expression can also evoke the corresponding emotion in perceivers (Berger, 1962; Bandura and Rosenthal, 1966; Hygge, 1976). Other research shows that mimicry can be involved in the detection of change in facial expressions of emotion. Specifically, evidence suggests that individuals detect changes in the facial expression of another person through the feedback, and perhaps change in subjective state, caused by facial mimicry (Zajonc et al., 1987; Wallbott, 1991; Niedenthal, 1992; see also Niedenthal and Showers, 1991). Facial mimicry can occur unconsciously and has for example been observed in response to subliminally presented pictures and in cortically blind patients (Dimberg et al., 2000; Tamietto et al., 2009).

The majority of emotion studies make use of prototypical static facial expressions in their experimental paradigms which in fact are fairly impoverished representations of facial expressions in real life. In reality, facial expressions are sometimes partly occluded and not fully visible and almost always paired with other expressive signals including prosody and body language that provide a context (for example, see Regenbogen et al., 2012). The perception of facial expressions of emotion is influenced by such context cues. Context, even when it needs to be ignored within an experimental task, can completely shift the emotional category recognized in a facial expression (Meeren et al., 2005; Righart and de Gelder, 2006; Van den Stock et al., 2007; Aviezer et al., 2008; Kret and de Gelder, 2013; Kret et al., 2013a,b). In addition, facial expressions are dynamic by nature, with varying intensity and ambiguous. For brevity I will not further discuss these aspects, although I think it is important to mention that dynamics in facial stimuli are especially important not only to enhance recognition or increase naturalness, but also to create the *impression of an interaction* in observers [a face that slowly starts to smile (video) might be perceived more as a reaction to the participant than an already smiling face (picture)].

**FIGURE 2:**
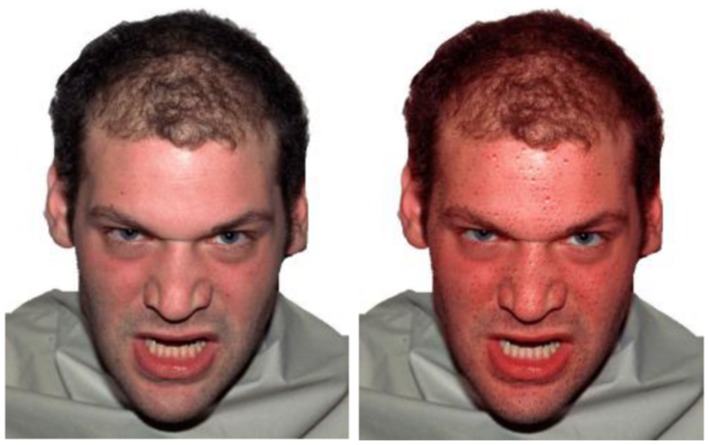
**Expressing anger**. The face on the right is reddened (except for the eyes and teeth) and shows drops of sweat. How these expressions of autonomic arousal impact on the perception of the emotional facial expression is not known.

In addition to facial actions, there are other ways for the face to reveal emotions than via its muscle movements. When highly emotional, our forehead may show sweat drops, our cheeks may blush, our eyes may tear and our pupils may dilate. All these automatic and autonomic reactions are not specific for one particular emotion and may also occur when in pain or during sport. Importantly, they may also happen during an emotional experience, and because they are much *harder to control* than our facial muscles, and *are visible to others*, they might add to the perceived intensity of a facial expression or even overrule the emotion signal the facial muscles try to reveal. Not much is known about how these autonomic reactions or signals impact on emotion perception and whether they can modulate or even change the perception of facial expressions, and provide a context. For example, it is possible that a person with an angry, reddened face and sweat pearls on his forehead is perceived as more intensely angry than a person without these signs of arousal. See **Figure 2**.

#### Facial Reddening

Facial redness is associated with the perfusion of the skin with oxygenated blood. Research shows that facial redness is sexually dimorphic (men are slightly redder than women; Edwards and Duntley, 1939; Frost, 1994), and is strongly androgen-dependent in men (Jeghers, 1944). Moreover, increased skin blood perfusion is related to health and is enhanced by physical training (Johnson, 1998) and reduced in different patient groups (Richardson, 1987; Panza et al., 1990; Charkoudian, 2003). Skin redness enhances the healthy appearance of faces, also in dark-skinned people (Stephen et al., 2009). A recent study examined whether one’s own positive and negative emotions affect how healthy we perceive our own face to appear as compared to how others faces appear. Participants reported on their own affective state and then judged their own face, either healthy (red-tinged) or unhealthy looking (green-tinged) against stranger’s faces. Results show that participants high in negative affectivity judged themselves as equivalent to less healthy looking versions of their own face and a stranger’s face (Mirams et al., 2014).

In addition to a healthy appearance, facial redness may also signal emotionality. Anger, for example, also increases the blood flow to the face (Drummond and Quah, 2001; Montoya et al., 2005). Stephen et al. (2012) asked a group of women to manipulate pictures of men’s faces to make them as attractive as possible. Women in this study made the skin tone redder and even added more red when asked to make the men look more dominant. However, it was also observed that the women associated very red faces with aggression. Clearly, these studies suggest that redness is associated with health, physical dominance and anger or aggression, and someone with reddened cheeks will likely be perceived as dominant, angry or aggressive. However, facial redness might also reflect shyness in the form of blushing.

Many socially anxious individuals are anxious about blushing and regard it as the cause of their social difficulties rather than as a symptom of them. Although there is a lot of research on why, when and who blushes (Leary et al., 1992), there is no research on how blushing is actually perceived by others. Dijk et al. (2011) examined whether blushing after a sociomoral transgression remediates trustworthiness in an interdependent context. In their study, participants played a computerized prisoner’s dilemma game with a virtual partner who defected in the second round of the game. After the defection, a picture of the opponent was shown, displaying a blushing (reddened) or a non-blushing face. In a subsequent trust-game, participants invested more money in the blushing opponent than in the non-blushing opponent. In addition, participants indicated that they trusted the blushing opponent more, that they expected a lower probability of future defeat, and judged the blushing opponent in general more positively.

Above research indicates that the redness of a face impacts on observers’ social judgments. However, exactly *how* a red face is interpreted (“shy blushing,” “angry,” more ambiguously “aroused” or simply “hot”) is unsure and deserves further investigation and experimental control. Another avenue for future research is to use dynamic morphs from normally colored to slightly reddened faces and to manipulate the redness of the face so that the observers get the impression that the reddening of the face is a reaction to them, as in social interaction. In addition, it would be interesting to test whether humans would mimic or synchronize with such changes in color in their own face, which could be predicted based on previous research showing that people synchronize their level of arousal (Levenson and Gottman, 1983; Feldman et al., 2011; Cooper et al., 2014).

### Emotions Shown by the Eyes

Among the many implicit cues that may inform assessments of someone’s internal state, the human eye region stands out as salient and powerful. Especially during short distance intimate interactions, both infants and adults focus on their interaction partner’s eyes, grasp emotion signals and follow gaze (Baron-Cohen, 1997; Driver et al., 1999; Farroni et al., 2002; Macrae et al., 2002).

#### Eyes and Gaze-Direction

The eyes are richly informative and important for understanding emotion and communicative intention of other individuals (Emery, 2000). Emotion-driven complex musculature contractions such as the raising of the eyebrows in fear enables observers to decode emotions from just the eye region (Baron-Cohen, 1997). The eye region captures more attention than other areas of the face in adults (Janik et al., 1978; Adolphs et al., 2005) as well as in infants (Haith et al., 1977) and this bias may reflect an innate predisposition (Argyle and Dean, 1965). In view of the importance of the eye region, one may predict that information from the eyes is robust such as to resist influence from the surrounding context. We often only see the eyes because items such as caps, hats, helmets, medical masks, beards or headdresses hide the rest of the face. Whether the perception of the expression of the eyes is sensitive to such visual context cues is a question that has hardly ever been asked.

There is some evidence that emotion categorization from the eye region is a process that is triggered automatically and unconsciously in a bottom–up fashion on the basis of the information available from the position of the eyebrows (Sadrô et al., 2003; Leppänen et al., 2008) and the visibility or display of eye white (Whalen et al., 2004). However, other research suggests that it is too early to rule out that context does play a role in the perception of expressions from the eyes. For example, Kret and de Gelder (2012) investigated how briefly presented angry, fearful, happy, and sad expressions were recognized when presented in different contexts including Islamic headdresses, a cap or a scarf. Results show that participants (all with a non-Islamic background) were better at recognizing fear from women wearing a niqāb as compared to from women wearing a cap and a shawl. An opposite effect was found for expressions of sadness and happiness. Furthermore, response patterns showed that ‘anger’ and ‘fear’ were more often chosen when the observed woman wore a niqāb as compared to a cap and a shawl. An opposite pattern was again found for the label ‘happy.’ Islamic cues triggered negative associations with the Islam, which influenced how emotions from the eyes were perceived. In line with the face literature this study shows that the recognition of emotional expressions from the eyes is also influenced by context.

Direct eye gaze captures and holds visual attention more efficiently than averted gaze and signals an expressor’s approach-avoidance behavioral tendency (Stern, 1977; von Grünau and Anston, 1995; Conty et al., 2010; Palanica and Itier, 2012; Böckler et al., 2014). Baron-Cohen (1995) states that this innate capacity to process gaze direction plays an important role in the development of a ‘Theory Of Mind.’

Gaze direction of an expressor also impacts on how observers perceive an expressed emotion. Adams and Kleck (2003) showed that expressions of anger and happiness were easier identified when presented with direct versus averted gaze, whereas fear and sadness expressions were more quickly labeled when presented with averted than with direct gaze. In a fear-conditioning experiment, Wieser et al. (2014) did not find an interaction between gaze direction and emotional expression either in terms of visually evoked steady-state potentials amplitude or affective ratings. This research underscores the importance of incorporating gaze direction in future work on facial expression perception.

Recent research has shown that social cognition including emotion perception is fundamentally different when we are engaged with others in real-time social interaction with eye-contact (‘online’ social cognition), rather than merely observing them (‘oﬄine’ social cognition; e.g., Pfeiffer et al., 2013; Schilbach et al., 2013). The time has come to study emotion perception during real interactions between two participants as the mechanisms underlying actual social interactions in real life are insufficiently understood.

#### Eyeblinks

Apart from social interest or attention that may be inferred from gaze cues, research suggests that humans are also sensitive to another’s eyeblinks. Humans spontaneously blink every few seconds. Eyeblinks are necessary to moisturize the eye, but occur more frequently than necessary. The spontaneous eye blink is considered to be a suitable ocular indicator for fatigue diagnostics (Stern et al., 1984) and reflects the influence of central nervous activation without voluntary manipulation (Blount, 1928; Ponder and Kennedy, 1928). Nakano et al. (2009) showed that spontaneous blinks synchronized between and within subjects when they viewed short video stories. Synchronized blinks occurred during scenes that required less attention such as at the conclusion of an action, during the absence of the main character, during a long shot and during repeated presentations of the same scene. In contrast, blink synchronization was not observed when subjects viewed a background video or when they listened to a story that was read aloud. The results suggest that humans share a mechanism for controlling the timing of blinks that optimizes the processing of critical information, in order to not miss out on important information while viewing a stream of visual events. In a next study, Nakano et al. (2013) show that eyeblinks are involved in attentional disengagement. Authors demonstrated that during cognitive load, eyeblinks momentarily activate the brain’s default-mode network, while deactivating the dorsal attention network. It is thus far unknown whether the observation of eyeblinks in another person influences how that person is perceived.

#### Tears

Crying is a typically and, as far as known, uniquely human form of emotional expression. This phenomenon is controlled by the sympathetic and the parasympathetic nervous system, the latter being responsible for the tears (Botelho, 1964). Recent research has offered several accounts of how tears may have become adaptive over the course of evolution. Two main functions of crying have been distinguished, namely (i) tension relief and promoting the recovery of psychological and physiological homeostasis, and (ii) communication. Thus, on the one hand crying may impact on psychobiological processes in the individual and facilitate the physiological and psychological recovery after distress but on the other hand it may elicit positive or negative reactions from the social environment (Hendriks and Vingerhoets, 2002). For example, Hill and Martin (1997) observed that crying female confederates were more sympathized with than non-crying confederates. The results of this study suggest that crying may communicate the need for emotional support and help and calls on empathy. Crying is very contagious. Even newborn children may spontaneously start crying when they hear other children cry (Simner, 1971). It has been argued that empathy is related to the capacity to react to and mimic emotional others. In line with this argument, Wiesenfeld et al. (1984) observed that highly empathic women were more likely to react with a corresponding facial reaction when observing crying infants.

Research has shown that tears are an important visual cue that add meaning to facial expressions. Provine et al. (2009) and Zeifman and Brown (2011) have demonstrated that tears are helpful to identify sadness and to perceive a need for help and comfort. Similarly, Balsters et al. (2013) show that tears increase the accurate recognition of sadness and also the extent to which the displayed crying persons were in need of social support. Interestingly, in this last study, images were shown for just 50 ms, demonstrating the strength of tears even at an early pre-attentive level.

One consequence of crying is that the eyes redden. Although research on the perception of sclera color exists, this is mostly focusing on perceived health, age and attractiveness. For example, faces with artificially reddened sclera are rated less attractive (Provine et al., 2011). Whether red eyes also impact on the perception of a facial expression is an open question.

#### The Perception of Another’s’ Pupil-Dilation

Pupil size is an interesting social signal because it cannot be controlled or faked, in contrast to features such as eye gaze and facial expression and reflects much more than changes in light, namely, our inner cognitive and affective state (Bradshaw, 1967). Precisely because pupil changes are unconscious, they provide a veridical reflection of the person’s inner state.

**FIGURE 3:**
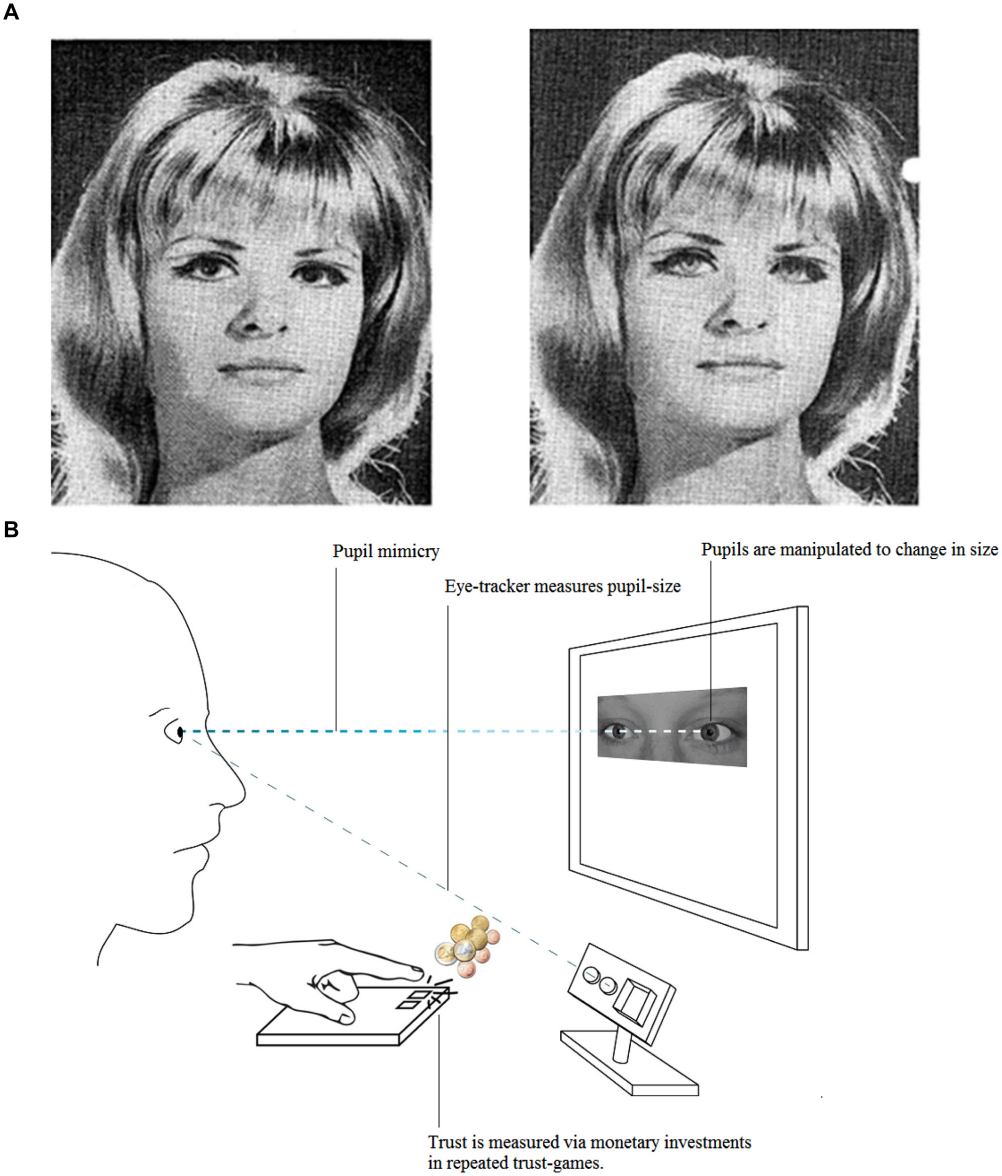
**Observed pupil size impacts on social evaluations. (A)** The woman on the left is judged positive and attractive, and the one on the right with small pupils, as cold and distant. Picture is taken from Hess (1975). **(B)** A partner with dilating pupils is better trusted than a partner with static or constricting pupils, especially when the participant (on the left) synchronizes his own pupil size with the observed partner. Visual representation of the experimental setup of the study by Kret et al. (2015).

Hess (1975) was the first to recognize the social potential of pupil dilation. In one study, he presented a group of men a series of pictures of which two showed an attractive young woman. One of them had been retouched to make the woman’s pupils larger and the other smaller than the original version. Interestingly, participant’s pupil response to the picture with the large pupils was larger than to the one with small pupils. Despite being unaware of the manipulation, participants liked the woman with the large pupils better, describing her as “more feminine,” “prettier,” and “softer” than the woman with small pupils (Hess, 1975; **Figure 3A**). In order to control for possible effects of luminance, in a later study, Hess created schematic eyes that consisted of a circle with a small, medium or large black dot in the middle. The circles were presented in isolation, in pairs with equally sized black dots, or with three of those in a row. Participants observed these stimuli whilst their pupil size was measured. Hess observed that both male and female participants showed the greatest pupil response to the ‘eye-like’ pair with the large black dot in the middle, an effect that could not be explained by luminance (Hess, 1975).

Later research replicated this early work and showed that pupillary changes are indeed picked up by observers and influence assessments: partners with large pupils are judged positive and attractive, and those with small pupils cold and distant (Demos et al., 2008; Amemiya and Ohtomo, 2012).

Kret et al. (2014) suggest that this positive association with large pupils is formed *through* pupil-mimicry. Within a close social interaction, mutual pupil dilation might indicate and reinforce social interest and liking. In their study, human and chimpanzee subjects viewed the eye region of both species. In this eye region, the stimulus pupils were manipulated to dilate or constrict dynamically, suggesting a response in view of the participant, as in an interaction. Results show that pupil-mimicry is not uniquely human, but has also been observed in chimpanzees. Even more interestingly, the phenomenon was restricted to within-species interactions (i.e. humans with humans, and chimpanzees with chimpanzees). It should be noted that the human participants in this study were mostly Japanese with pupils hardly distinguishable from their dark irises. Even in them, pupil-mimicry occurred, which is in line with the great importance of pupil size in the Japanese culture. As in Disney figures, good and bad cartoon characters in Japanese Manga are characterized by large and small pupils respectively.

The study by Kret et al. (2014) suggests that apart from passively reflecting inner states, pupils have a social signaling function and provide a basic form of communication between members of one’s own species. A more recent study further supports that idea. Kret et al. (2015; **Figure 3B**) observed that participants not only synchronized their pupil size with the pupil size of their virtual partner, but that this also helped them to trust that partner. More precisely, in their study, they used eye-images of Dutch and Japanese students and presented these to Dutch students. The pupils within these images dilated, constricted, or remained static. Whereas pupil dilation mimicry was amplified in interactions with the Dutch partners (who were considered closer to the in-group than the Japanese partners), pupil-constriction mimicry was stronger with the Japanese partners. Interestingly, a link was observed between pupil dilation mimicry and trust in Dutch, but not Japanese partners. Whether pupil-mimicry impacts on real interactions is unsure; pupils are prone to changes in ambient light and as mentioned earlier, are not equally visible in all individuals or circumstances. However, the fact that pupils have such profound effects in lab studies with virtual partners demonstrates their large potential in future live interaction studies with two participants.

## Conclusion

During social interactions, humans willingly and unwillingly express their emotional state through facial muscle movements but also via other channels including pupil-dilation, eyeblinks and blushing. Over the course of human evolution, it became adaptive to not only perceive and be aware of emotions in one self, but also to process emotions including these implicit cues of others. Such perceptual abilities insure smooth interpersonal cooperation, for example by permitting the monitoring of expressions of fear and facilitating quick actions in response to environmental threat. In the past century, research on this topic has accumulated steadily and shown that humans, from early age on, respond to the emotions of others, empathize with them, and are very good at distinguishing between different expressions of emotion (i.e., Izard, 1971; Ekman and Friesen, 1982). The majority of this research has focused on the perception of pictures of faces showing prototypical, intense and unambiguous emotional expressions. Research has for example shown that humans mimic facial expressions (Dimberg, 1982) and according to the theory of emotional contagion, this might even improve emotion recognition (Hatfield et al., 1994).

At the beginning of this article, I noted the importance of context in the perception of emotions from the face. Emotions as displayed in the face are perceived in the context of the person’s body posture and the social emotional scene context (Kret and de Gelder, 2010). Clearly, the studies that did take these factors into account show that the percept of a face can change as a function of these contextual factors (Meeren et al., 2005; Righart and de Gelder, 2006; Van den Stock et al., 2007; Aviezer et al., 2008; Kret et al., 2013a,b). Also, emotions seen from just the eye region appear different, depending on what is visible around the eyes such as various types of clothing including caps, scarves and Islamic headdresses (Kret and de Gelder, 2012). My argument that autonomic expressions of arousal such as pupil dilation are less prone to cognitive control than facial expressions does not imply that they are not sensitive to contextual factors. On the contrary, pupil-mimicry for example, a phenomenon individuals are absolutely unaware of when doing it, is enhanced in interactions with members of an in-group (other humans and own culture) as compared to members of an out-group (chimpanzees and other cultural and ethnical background; Kret et al., 2014, 2015). I would like to argue that facial expressions of emotion beyond facial muscle actions can provide an additional context for the interpretation of emotional expressions.

When experiencing an emotion, it is not just the facial muscles that communicate that to other individuals. The current article is not the first call for studying ‘emotion-signaling sources’ beyond the well-known facial muscle movements. Other research has for example stressed the importance of studying the perception of bodily expressions (Dittrich et al., 1996; Pollick et al., 2001; Kret et al., 2011a,b,c, 2013a,b; Sokolov et al., 2011; Krüger et al., 2013). Although facial muscle actions can be unconscious, it is possible to control or regulate them via ‘top–down’ cognitive processes, yet our verical emotion often speaks through our body (for a review, see de Gelder et al., 2010). While fully acknowledging the importance of that work, I here would like to stress that also within a face there are more verical emotion signals than the signals sent by the facial muscles. Instead, autonomic expressions of arousal are much harder to control and more driven in a bottom-up fashion. For that reason, autonomic expressions might provide implicit insight into another’s experienced emotions, and might impact on the perceived intensity or genuineness of the expressed emotion.

To conclude, psychophysiological research has shown that the perception of emotional expressions in another individual triggers autonomic reactions that are for example measurable via electrodes measuring skin conductance (sweat), eye-tracking equipment (fixated attention and pupil dilation), (i.e. Bradley et al., 2008) or thermal imaging (to measure facial heating; for a review, see Ioannou et al., 2014). All these measures, these dependent variables, are in principle *visible* to others, and could serve as independent variables, possibly impacting on the total percept of a person and his emotional expression, its intensity and/or genuineness. The shedding of tears in sadness or joy, the reddening of the face and the pearls of sweat on ones forehead in anger or love, the blushing of the face and averting of gaze in embarrassment or shame, the dilating pupils indicating social interest and trust, happiness or stress are just examples of emotion signals that have received very little attention in the literature. Studying emotion perception beyond the face muscles including the perception of autonomic signals and the synchronization therewith is a promising avenue for future research.

## Conflict of Interest Statement

The author declares that the research was conducted in the absence of any commercial or financial relationships that could be construed as a potential conflict of interest.

## References

[B1] Adams R. B. Jr. Kleck R. E. (2003). Perceived gaze direction and the processing of facial displays of emotion. *Psychol. Sci.* 14 644–647. 10.1046/j.0956-7976.2003.psci_1479.x 14629700

[B2] Adolphs R. Gosselin F. Buchanan T. W. Tranel D. Schyns P. Damasio A. R. (2005). A mechanism for impaired fear recognition after amygdala damage. *Nature* 433 68–72. 10.1038/nature03086 15635411

[B3] Amemiya S. Ohtomo K. (2012). Effect of the observed pupil size on the amygdala of the beholders. *Soc. Cogn. Affect. Neurosci.* 7 332–341. 10.1093/scan/nsr013 21421732 PMC3304484

[B4] Argyle M. Dean J. (1965). Eye-contact, distance and affiliation. *Sociometry* 28 289–304. 10.2307/2786027 14341239

[B5] Aviezer H. Hassin R. R. Ryan J. Grady C. Susskind J. Anderson A. (2008). Angry, disgusted, or afraid? Studies on the malleability of emotion perception. *Psychol. Sci.* 19 724–732. 10.1111/j.1467-9280.2008.02148.x 18727789

[B6] Balsters M. J. H. Krahmer E. J. Swerts M. G. J. Vingerhoets A. J. J. M. (2013). Emotional tears facilitate the recognition of sadness and the perceived need for social support. *Evol. Psychol.* 11 148–158. 23531802 10.1177/147470491301100114 PMC10480939

[B7] Bandura A. Rosenthal T. L. (1966). Vicarious classical conditioning as a function of arousal level. *J. Pers. Soc. Psychol.* 3 54–62. 10.1037/h0022639 5902077

[B8] Baron-Cohen S. (1995). *Theory of Mind and Face-Processing: How do They Interact in Development and Psychopathology?* New York: Wiley.

[B9] Baron-Cohen S. (1997). *Mindblindness: An Essay on autism and Theory of Mind*. Cambridge, MA: MIT Press.

[B10] Berger S. M. (1962). Conditioning through vicarious instigation. *Psychol. Rev.* 69 450–466. 10.1037/h0046466 13867454

[B11] Biehl M. Matsumoto D. Ekman P. Hearn V. Heider K. Kudoh T. (1997). Matsumoto and Ekman’s Japanese and Caucasian facial expressions of emotion (JACFEE): reliability data and cross-national differences. *J. Nonverbal Behav.* 21 3–21. 10.1023/A:1024902500935

[B12] Blount W. P. (1928). Studies of the movements of the eyelids of animals: blinking. *Q. J. Exp. Physiol.* 18 111–125. 10.1113/expphysiol.1927.sp000426

[B13] Böckler A. van der Wel R. P. R. D. Welsh T. N. (2014). Catching eyes: effects of social and nonsocial cues on attention capture. *Psychol. Sci.* 25 720–727. 10.1177/0956797613516147 24398595

[B14] Botelho S. (1964). Tears and the lacrimal gland. *Sci. Am.* 221 78–86. 10.1038/scientificamerican1064-78 14216948

[B15] Bradley M. M. Miccoli L. Escrig M. A. Lang P. J. (2008). The pupil as a measure of emotional arousal and autonomic activation. *Psychophysiology* 45 602–607. 10.1111/j.1469-8986.2008.00654.x 18282202 PMC3612940

[B16] Bradshaw J. (1967). Pupil size as a measure of arousal during information processing. *Nature* 216 515–516. 10.1038/216515a0 6057275

[B17] Bush L. K. Barr C. L. McHugo G. J. Lanzetta J. T. (1989). The effects of facial control and facial mimicry on subjective reactions to comedy routines. *Motiv. Emot.* 13 31–52. 10.1007/BF00995543

[B18] Calvo M. G. Fernández-Martín A. Nummenmaa L. (2012). Perceptual, categorical, and affective processing of ambiguous smiling facial expressions. *Cognition* 125 373–393. 10.1016/j.cognition.2012.07.021 22939734

[B19] Charkoudian N. (2003). Skin blood flow in adult human thermoregulation: how it works, when it does not, and why. *Mayo Clin. Proc.* 78 603–612. 10.4065/78.5.603 12744548

[B20] Conty L. Gimmig D. Belletier C. George N. Huguet P. (2010). The cost of being watched: stroop interference increases under concomitant eye contact. *Cognition* 115 133–139. 10.1016/j.cognition.2009.12.005 20070959

[B21] Cooper E. A. Garlick J. Featherstone E. Voon V. Singer T. Critchley H. D. (2014). You turn me cold: evidence for temperature contagion. *PLoS ONE* 9:e116126 10.1371/journal.pone.0116126 PMC4281213 25551826

[B22] de Gelder B. Teunisse J. P. Benson P. (1997). Categorical perception of facial expressions: categories and their internal structure. *Cogn. Emot.* 11 1–23. 10.1080/026999397380005

[B23] de Gelder B. van den Stock J. Meeren H. K. M. Sinke C. B. A. Kret M. E. Tamietto M. (2010). Standing up for the body. Recent progress in uncovering the networks involved in processing bodies and bodily expressions. *Neurosci. Biobehav. Rev.* 34 513–527. 10.1016/j.neubiorev.2009.10.008 19857515

[B24] Demos K. E. Kelley W. M. Ryan S. L. Davis F. C. Whalen P. J. (2008). Human amygdala sensitivity to the pupil size of others. *Cereb. Cortex* 18 2729–2734. 10.1093/cercor/bhn034 18372291 PMC2583162

[B25] Dijk C. Koenig B. Ketelaar T. de Jong P. J. (2011). Saved by the blush: being trusted despite defecting. *Emotion* 11 313–319. 10.1037/a0022774 21500900

[B26] Dimberg U. (1982). Facial reactions to facial expressions. *Psychophysiology* 19 643–647. 10.1111/j.1469-8986.1982.tb02516.x 7178381

[B27] Dimberg U. Thunberg M. Elmehed K. (2000). Unconscious facial reactions to emotional facial expressions. *Psychol. Sci.* 11 86–89. 10.1111/1467-9280.00221 11228851

[B28] Dittrich W. H. Troscianko T. Lea S. E. Morgan D. (1996). Perception of emotion from dynamic point-light displays represented in dance. *Perception* 25 727–738. 10.1068/p250727 8888304

[B29] Driver J. Davis G. Ricciardelli P. Kidd P. Maxwell E. Baron-Cohen S. (1999). Gaze perception triggers reflexive visuospatial orienting. *Vis. Cogn.* 6 509–540. 10.1080/135062899394920

[B30] Drummond P. D. Quah S. H. (2001). The effect of expressing anger on cardiovascular reactivity and facial blood flow in Chinese and Caucasians. *Psychophysiology* 38 190–196. 10.1111/1469-8986.3820190 11347864

[B31] Edwards E. A. Duntley S. Q. (1939). The pigments and color of living human skin. *Am. J. Anat.* 65 1–33. 10.1002/aja.1000650102

[B32] Ekman P. Friesen W. V. (1978). *Facial Action Coding System: a Technique for the Measurement of Facial Movement*. Palo Alto: Consulting Psychologists Press.

[B33] Ekman P. Friesen W. V. (1982). Felt, false, and miserable smiles. *J. Nonverbal Behav.* 6 238–252. 10.1007/BF00987191

[B34] Emery N. J. (2000). The eyes have it: the neuroethology, function and evolution of social gaze. *Neurosci. Biobehav. Rev.* 24 581–604. 10.1016/S0149-7634(00)00025-7 10940436

[B35] Farroni T. Csibra G. Simion F. Johnson M. H. (2002). Eye contact detection in humans from birth. *Proc. Natl. Acad. Sci. U.S.A.* 99 9602–9605. 10.1073/pnas.152159999 12082186 PMC123187

[B36] Feldman R. Magori-Cohen R. Galili G. Singer M. Louzoun Y. (2011). Mother and infant coordinate heart rhythms through episodes of interaction synchrony. *Infant Behav. Dev.* 34 569–577. 10.1016/j.infbeh.2011.06.008 21767879

[B37] Frost P. (1994). Preferences for darker faces in photographs at different phases of the menstrual cycle: preliminary assessment of evidence for a hormonal relationship. *Percept. Motor Skills* 79 507–514. 10.2466/pms.1994.79.1.507 7808889

[B38] Haith M. M. Bergman T. Moore M. J. (1977). Eye contact and face scanning in early infancy. *Science* 198 853–855. 10.1126/science.918670 918670

[B39] Hatfield E. Cacioppo J. Rapson R. L. (1994). *Emotional Contagion*. New York: Cambridge University Press.

[B40] Hendriks M. C. P. Vingerhoets A. J. J. M. (2002). Crying: is it beneficial for one’s well-being? *Int. Congr. Ser.* 1241 361–365. 10.1016/S0531-5131(02)00642-8

[B41] Hess E. H. (1975). The role of pupil size in communication. *Sci. Am.* 233 110–112. 10.1038/scientificamerican1175-110 1188340

[B42] Hill P. Martin R. B. (1997). Empathic weeping, social communication, and cognitive dissonance. *J. Soc. Clin. Psychol.* 16 299–322. 10.1521/jscp.1997.16.3.299

[B43] Hygge S. (1976). *Emotional and Electrodermal Reactions to the Suffering of Another: Vicarious Instigation and Vicarious Classical Conditioning.* Stockholm: Almqvist & Wiksell.

[B44] Ioannou S. Gallese V. Merla A. (2014). Thermal infrared imaging in psychophysiology: potentialities and limits. *Psychophysiology* 51 951–963. 10.1111/psyp.12243 24961292 PMC4286005

[B45] Izard C. (1971). *The Face of Emotion*. New York: Appleton-Century-Crofts.

[B46] Janik S. W. Wellens A. R. Goldberg M. L. Dell’Osso L. F. (1978). Eyes as the center of focus in the visual examination of human faces. *Percept. Motor Skills* 47 857–858. 10.2466/pms.1978.47.3.857 740480

[B47] Jeghers H. (1944). Pigmentation of the skin. *New Engl. J. Med*. 231 88–100. 10.1056/NEJM194407202310304

[B48] Johnson J. M. (1998). Physical training and the control of skin blood flow. *Med. Sci. Sports Exerc*. 30 382–386. 10.1097/00005768-199803000-00007 9526883

[B49] Kret M. E. de Gelder B. (2010). Social context influences recognition of bodily expressions. *Exp. Brain Res.* 203 169–180. 10.1007/s00221-010-2220-8 20401473 PMC2862946

[B50] Kret M. E. de Gelder B. (2012). Islamic headdress influences how emotion is recognized from the eyes. *Front. Psychol.* 3:110 10.3389/fpsyg.2012.00110 PMC3322610 22557983

[B51] Kret M. E. de Gelder B. (2013). When a smile becomes a fist: the perception of facial and bodily expressions of emotion in violent offenders. *Exp. Brain Res.* 228, 399–410. 10.1007/s00221-013-3557-6 23828232 PMC3710410

[B52] Kret M. E. Fischer A. H. de Dreu C. K. W. (2015). Pupil-mimicry correlates with trust in in-group partners with dilating pupils. *Psychol. Sci*. 16 50. 10.1177/0956797615588306 26231910

[B53] Kret M. E. Pichon S. Grèzes J. de Gelder B. (2011a). Similarities and differences in perceiving threat from dynamic faces and bodies: an fMRI study. *NeuroImage* 54 1755–1762. 10.1016/j.neuroimage.2010.08.012 20723605

[B54] Kret M. E. Pichon S. Grèzes J. de Gelder B. (2011b). Men fear other men most: gender specific brain activations in perceiving threat from dynamic faces and bodies - an fMRI study. *Front. Psychol.* 2:3 10.3389/fpsyg.2011.00003 PMC3111446 21713131

[B55] Kret M. E. Denollet J. Grèzes J. de Gelder B. (2011c). The role of negative affectivity and social inhibition in perceiving social threat: an fMRI study. *Neuropsychologia* 49 1187–1193. 10.1016/j.neuropsychologia.2011.02.007 21315749

[B56] Kret M. E. Ploeger A. (2015). Emotion processing deficits: a liability spectrum providing insight into comorbidity of mental disorders. *Neurosci. Biobehav. Rev.* 52 153–171. 10.1016/j.neubiorev.2015.02.011 25725415

[B57] Kret M. E. Roelofs K. Stekelenburg J. J. de Gelder B. (2013a). Emotional signals from faces, bodies and scenes influence observers’ face expressions, fixations and pupil size. *Front. Hum. Neurosci.* 7:810 10.3389/fnhum.2013.00810 PMC3866922 24391567

[B58] Kret M. E. Stekelenburg J. J. Roelofs K. de Gelder B. (2013b). Perception of face and body expressions using electromyography, pupillometry and gaze measures. *Front. Psychol.* 4:28 10.3389/fpsyg.2013.00028 PMC3567353 23403886

[B59] Kret M. E. Tomonaga M. Matsuzawa T. (2014). Chimpanzees and humans mimic pupil-size of conspecifics. *PLoS ONE* 9:e104886 10.1371/journal.pone.0104886 PMC4139319 25140998

[B60] Krüger S. Sokolov A. N. Enck P. Krägeloh-Mann I. Pavlova M. A. (2013). Emotion through locomotion: gender impact. *PLoS ONE* 8:e81716 10.1371/journal.pone.0081716 PMC3838416 24278456

[B61] Leary M. R. Britt T. W. Cutlip W. D. Templeton J. L. (1992). Social blushing. *Psychol. Bull.* 112 446–460. 10.1037/0033-2909.112.3.446 1438638

[B62] Leppänen J. M. Hietanen J. K. Koskinen K. (2008). Differential early ERPs to fearful versus neutral facial expressions: a response to the salience of the eyes? *Biol. Psychol.* 78 150–158. 10.1016/j.biopsycho.2008.02.002 18359141

[B63] Levenson R. W. Gottman J. M. (1983). Marital interaction: physiological linkage and affective exchange. *J. Pers. Soc. Psychol.* 45 587–597. 10.1037/0022-3514.45.3.587 6620126

[B64] Lipp O. V. Price S. M. Tellegen C. L. (2009). No effect of inversion on attentional and affective processing of facial expressions. *Emotion* 9 248–259. 10.1037/a0014715 19348536

[B65] Macrae C. N. Hood B. M. Milne A. B. Rowe A. C. Mason M. F. (2002). Are you looking at me? Gaze and person perception. *Psychol. Sci.* 13 460–464. 10.1111/1467-9280.00481 12219814

[B66] Maurer D. Le Grand R. Mondloch C. J. (2002). The many faces of configural processing. *Trends Cogn. Sci.* 6 255–260. 10.1016/S1364-6613(02)01903-4 12039607

[B67] Meeren H. K. M. van Heijnsbergen C. de Gelder B. (2005). Rapid perceptual integration of facial expression and emotional body language. *Proc. Natl. Acad. Sci. U.S.A.* 102 16518–16523. 10.1073/pnas.0507650102 16260734 PMC1283446

[B68] Mirams L. Poliakoff E. Zandstra E. H. Hoeksma M. Thomas A. El-Deredy W. (2014). Feeling bad and looking worse: negative affect is associated with reduced perceptions of face-healthiness. *PLoS ONE* 9:e107912 10.1371/journal.pone.0107912 PMC4178040 25259802

[B69] Montoya P. Campos J. J. Schandry R. (2005). See red? Turn pale? Unveiling emotions through cardiovascular and hemodynamic changes. *Span. J. Psychol.* 8 79–85. 10.1017/S1138741600004984 15875460

[B70] Nakano T. Kato M. Morito Y. Itoi S. Kitazawa S. (2013). Blink-related momentary activation of the default mode network while viewing videos. *Proc. Natl. Acad. Sci. U.S.A.* 110 702–706. 10.1073/pnas.1214804110 23267078 PMC3545766

[B71] Nakano T. Yamamoto Y. Kitajo K. Takahashi T. Kitazawa S. (2009). Synchronization of spontaneous eyeblinks while viewing video stories. *Proc. R. Soc. B Biol. Sci.* 276 3635–3644. 10.1098/rspb.2009.0828 PMC2817301 19640888

[B72] Niedenthal P. M. (1992). “Affect and social perception: on the psychological validity of rose-colored glasses,” in *Perception Without Awareness*, eds Bornstein R. F. Pittman T. S. (New York: Guilford Press).

[B73] Niedenthal P. M. Showers C. (1991). “The perception and processing of affective information and its influences on social judgment,” in *Emotion and Social Judgment*, ed. Fogas J. P. (Oxford: Pergamon).

[B74] Palanica A. Itier R. J. (2012). Attention capture by direct gaze is robust to context and task demands. *J. Nonverbal. Behav.* 36 123–134. 10.1007/s10919-011-0128-z 24976664 PMC4072644

[B75] Panza J. A. Quyyimi A. A. Brush J. R. Epstein S. E. (1990). Abnormal endothelium-dependent vascular relaxation in patients with essential hypertension. *N. Engl. J. Med*. 323 22–27. 10.1056/NEJM199007053230105 2355955

[B76] Pfeiffer U. J. Vogeley K. Schilbach L. (2013). From gaze cueing to dual eye-tracking: novel approaches to investigate the neural correlates of gaze in social interaction. *Neurosci. Biobehav. Rev.* 37(Pt 2), 2516–2528. 10.1016/j.neubiorev.2013.07.017 23928088

[B77] Pollick F. E. Paterson H. M. Bruderlin A. Sanford A. J. (2001). Perceiving affect from arm movement. *Cognition* 82 B51–B61. 10.1016/S0010-0277(01)00147-0 11716834

[B78] Ponder E. Kennedy W. P. (1928). On the act of blinking. *Q. J. Exp. Physiol.* 18 89–110. 10.1113/expphysiol.1927.sp000433

[B79] Provine R. R. Cabrera M. O. Brocato N. W. Krosnowski K. A. (2011). When the whites of the eyes are red: a uniquely human cue. *Ethology* 117 395–399. 10.1111/j.1439-0310.2011.01888.x

[B80] Provine R. R. Krosnowski K. A. Brocato N. W. (2009). Tearing: breakthrough in human emotional signaling. *Evol. Psychol.* 7 52–56.

[B81] Regenbogen C. Schneider D. A. Finkelmeyer A. Kohn N. Derntl B. Kellermann T. (2012). The differential contribution of facial expressions, prosody, and speech content to empathy. *Cogn. Emot.* 26 995–1014. 10.1080/02699931.2011.631296 22214265

[B82] Richardson D. (1987). Effects of tobacco smoke inhalation on capillary blood flow in human skin. *Arch. Environ. Health* 42 19–25. 10.1080/00039896.1987.9935790 3566346

[B83] Righart R. de Gelder B. (2006). Context influences early perceptual analysis of faces. An electrophysiological study. *Cereb. Cortex* 16 1249–1257. 10.1093/cercor/bhj066 16306325

[B84] Sadrô J. Jarudi I. Sinha P. (2003). The role of eyebrows in face recognition. *Perception* 32 285–293. 10.1068/p5027 12729380

[B85] Savage R. A. Lipp O. V. (2014). The effect of face inversion on the detection of emotional faces in visual search. *Cogn. Emot.* 17 1–20. 10.1080/02699931.2014.958981 25229360

[B86] Schilbach L. Timmermans B. Reddy V. Costall A. Bente G. Schlicht T. (2013). Toward a second-person neuroscience. *Behav. Brain Sci.* 36 393–462. 10.1017/S0140525X12000660 23883742

[B87] Simner M. L. (1971). Newborns’ response to the cry of another infant. *Dev. Psychol.* 5 136–150. 10.1037/h0031066

[B88] Sokolov A. A. Krüger S. Enck P. Krägeloh-Mann I. Pavlova M. A. (2011). Gender affects body language reading. *Front. Psychol.* 2:16 10.3389/fpsyg.2011.00016 PMC3111255 21713180

[B89] Stephen I. D. Coetzee V. Law Smith M. J. Perrett D. I. (2009). Skin blood perfusion and oxygenation color affect perceived human health. *PLoS ONE* 4:e5083 10.1371/journal.pone.0005083 PMC2659803 19337378

[B90] Stephen I. D. Oldham F. H. Perrett D. I. Barton R. A. (2012). Redness enhances perceived aggression, dominance and attractiveness in men’s faces. *Evol. Psychol.* 10 562–572. 22947678 10.1177/147470491201000312 PMC10426935

[B91] Stern D. N. (1977). *The First Relationship: Infant and Mother*. Cambridge, MA: Harvard University Press.

[B92] Stern J. A. Walrath L. C. Goldstein R. (1984). The endogenous eyeblink. *Psychophysiology* 21 22–33. 10.1111/j.1469-8986.1984.tb02312.x 6701241

[B93] Tamietto M. Castelli L. Vighetti S. Perozzo P. Geminiani G. Weiskrantz L. (2009). Unseen facial and bodily expressions trigger fast emotional reactions. *Proc. Natl. Acad. Sci. U.S.A.* 106 17661–17666. 10.1073/pnas.0908994106 19805044 PMC2764895

[B94] Tanaka J. W. Farah M. J. (1993). Parts and wholes in face recognition. *Q. J. Exp. Psychol. A.* 46 225–245. 10.1080/14640749308401045 8316637

[B95] Van den Stock J. Righart R. de Gelder B. (2007). Body expressions influence recognition of emotions in the face and voice. *Emotion* 7 487–494. 10.1037/1528-3542.7.3.487 17683205

[B96] von Grünau M. Anston C. (1995). The detection of gaze direction: a stare-in-the-crowd effect. *Perception* 24 1297–1313. 10.1068/p241297 8643334

[B97] Wallbott H. G. (1991). Recognition of emotion from facial expression via imitation? Evidence for an old theory. *Br. J. Soc. Psychol.* 30 207–219. 10.1111/j.2044-8309.1991.tb00939.x 1933146

[B98] Whalen P. J. Kagan J. Cook R. G. Davis F. C. Kim H. Polis S. (2004). Human amygdala responsivity to masked fearful eye whites. *Science* 306 2061–2061. 10.1126/science.1103617 15604401

[B99] Wiesenfeld A. R. Whitman P. B. Malatesta C. Z. (1984). Individual differences among adult women in sensitivity to infants: evidence in support of an empathy concept. *J. Pers. Soc. Psychol.* 46 118–124. 10.1037/0022-3514.46.1.118 6694055

[B100] Wieser M. J. Miskovic V. Rausch S. Keil A. (2014). Different time course of visuocortical signal changes to fear-conditioned faces with direct or averted gaze: a ssVEP study with single-trial. *Neuropsychologia* 62 101–110. 10.1016/j.neuropsychologia.2014.07.009 25050854

[B101] Yin R. K. (1969). Looking at upside-down faces. *J. Exp. Psychol.* 81 141–145. 10.1037/h0027474

[B102] Zajonc R. B. Adelmann P. K. Murphy S. T. Niedenthal P. M. (1987). Convergence in the physical appearance of spouses. *Motiv. Emot.* 11 335–346. 10.1007/BF00992848

[B103] Zeifman D. M. Brown S. A. (2011). Age-related changes in the signal value of tears. *Evol. Psychol.* 9 313–324. 22947977 10.1177/147470491100900304 PMC10426848

